# Obstructive Sleep Apnea and Alzheimer’s Disease in Down Syndrome

**DOI:** 10.1002/alz.092772

**Published:** 2025-01-09

**Authors:** Dasoo Milton Yoon, Emily K Schworer, Victoria L Fleming, Benjamin L Handen, Jamie C Peven, David T Plante, Sigan L Hartley

**Affiliations:** ^1^ University of Wisconsin‐Madison, Madison, WI USA; ^2^ Waisman Center, University of Wisconsin‐Madison, Madison, WI USA; ^3^ University of Pittsburgh, Pittsburgh, PA USA; ^4^ University of Wisconsin School of Medicine and Public Health, Madison, WI USA

## Abstract

**Background:**

The present study examined OSA using an objective home sleep test in 81 adults with DS (aged 25 ∼ 61 years) and evaluated associations between sleep‐disordered breathing problems and biomarkers of AD pathology (PET Aβ and tau) and symptomology (cognitive performance and depressed mood).

**Method:**

As part of the ABC‐DS study, participants completed a 2.5‐hour battery of cognitive measures and underwent MRI and PET imaging scans and a blood draw. Study partners completed informant measures to report on the participant’s sociodemographics, dementia symptoms, and mood. As part of the lifestyle auxiliary study, the adult with DS wore the WatchPAT 300 device for one night to assess sleep‐disordered breathing and caregivers reported on sleep habits and any sleep‐related conditions or treatments. The adults with DS and study partners earned up to $200 for completion of ABC‐DS study activities and $50 for completion of WatchPAT study activities.

**Result:**

Of the 81 participants with valid sleep data, 67 (83%) screened positive for OSA based on the WatchPAT data. When considering pAHI, 25 (31%) had mild OSA, 19 (24%) had moderate OSA, and 22 (27%) had severe OSA. In total, 25 of the 39 (64%) participants without a medical diagnosis of OSA screened positive for OSA based on the WatchPAT. A chi‐square test indicated no significant difference in OSA severity level (none, mild, moderate, severe) between participants who did versus did not have a medical diagnosis of OSA (𝝌2 = 1.648, p = .648). Main findings between sleep and biomarker variables included PET Aβ being significantly associated with mean of desaturations, wake state, and total sleep time, while tau being significantly associated with % of REM Sleep, minimum oxygen level, wake state, and number of wakes.

**Conclusion:**

Findings indicate there is a high prevalence of sleep problems and OSA in adults with DS. There were also significant correlations between the presence and severity of OSA‐related sleep problems with higher PET Aβ and tau levels, as well as more AD‐related cognitive impairments and worse mood.

